# Cooperative Surface‐Particle Catalysis: The Role of the “Active Doughnut” in Catalytic Oxidation

**DOI:** 10.1002/cctc.201701819

**Published:** 2018-02-16

**Authors:** Thierry K. Slot, David Eisenberg, Gadi Rothenberg

**Affiliations:** ^1^ Van't Hoff Institute for Molecular Sciences University of Amsterdam Science Park 904 Amsterdam 1098 XH The Netherlands; ^2^ Current address: Schulich Faculty of Chemistry Technion-Israel Institute of Technology Haifa 3200003 Israel

**Keywords:** active volume, catalyst design, catalytic oxidation, particle-surface reactions, tandem reactions

## Abstract

We consider the factors that govern the activity of bifunctional catalysts comprised of active particles supported on active surfaces. Such catalysts are interesting because the adsorption and diffusion steps, which are often discounted in “conventional” catalytic scenarios, play a key role here. We present an intuitive model, the so‐called “active doughnut” concept, defining an active catalytic region around the supported particles. This simple model explains the role of adsorption and diffusion steps in cascade catalytic cycles for active particles supported on active surfaces. The concept has two important practical implications. First, the reaction rate is no longer proportional to the number of active sites, but rather to the number of “*communicative”* active sites—those available to the reaction intermediates during their respective lifetimes. Second, it generates an important testable prediction concerning the dependence of the total reaction rate on the particle size. With these tools at hand, we examine six experimental examples of catalytic oxidation from the literature, and show that the active doughnut concept gives valuable insight even when detailed mechanistic information is hard to come by.

## Introduction

Tandem reactions, in which two or more catalytic cycles are combined into one synthetic operation, have gained much attention recently.^1^, ^2^, ^3^ This reflects a shift in synthetic chemistry towards more complex systems, where elements of classic organic synthesis, biosynthesis, homogeneous catalysis and heterogeneous catalysis are combined into efficient one‐pot processes.^2^, ^4^ The increased complexity mimics natural systems, with the ultimate goal of combining the benefits of classical solid catalysts (easy separation and high stability) with those of biocatalysis and homogeneous catalysis (high product selectivity and mild reaction conditions).^5^, ^6^, ^7^

Designing such tandem catalysts is challenging, because per definition they must include a transition step between the two catalytic sites. This can complicate things compared to the classic Langmuir‐Hinshelwood model, where the steps of diffusion and adsorption/desorption are often discounted, especially in cases where the chemical reaction at the active site is rate‐determining. If a tandem system comprises two separate cycles (for example an acid‐catalysed reaction at one site followed by a base‐catalysed reaction at another^8^), then the classical model suffices. But when a catalytic cycle requires both sites, the diffusion of short‐lived intermediates between these sites cannot be ignored.^9^

**Figure 1:**
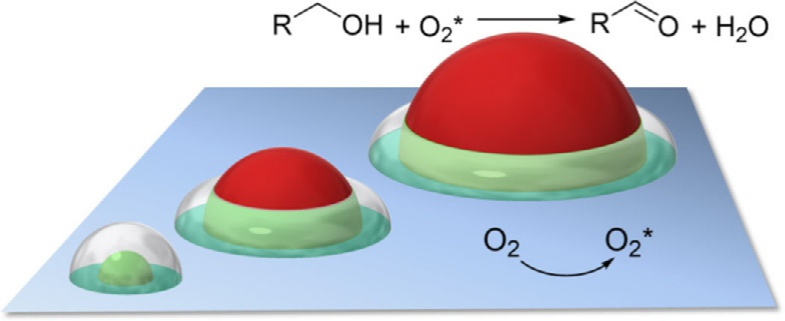
Cartoon of the cooperative action between a particle and a surface illustrating the “active doughnut” concept. Dioxygen is activated at the nitrogen‐doped carbon surface, and the activated oxygen species then travels to the metal oxide particle and reacts with an alcohol that has adsorbed there. Reaction conditions: 1 mmol R−OH, 20 mg catalyst, 5 mL ethanol, 1 atm O_2_, 80 °C, 16 h.

The simplest configuration of such a catalyst is an active particle on an active surface. The particle and the surface are each responsible for a part of the catalytic cycle, and active intermediates must travel between the two “sites”. The best way to illustrate this is by looking at an experimental example: Figure 1 shows the catalytic oxidation of alcohols with molecular oxygen in the presence of metal oxide particles on nitrogen‐doped carbon.^10^ Here, the nitrogen‐doped carbon surface activates incoming dioxygen molecules. These short‐lived active oxygen species then diffuse to the metal oxide particles, which catalyse the subsequent oxidation of alcohols. Common sense tells us that there must be a volume around each catalytic particle where the majority of the reactions occur. We call this toroidal volume the “active doughnut”.

In this concept paper, we propose a theoretical framework for describing the physical space in which two catalytic sites communicate. In particular, we focus on catalytically active *particles* dispersed on a catalytically active *surface*. The volume of interaction in such systems is toroidal, so we dub it the “active doughnut”. We first develop the concept based on theoretical considerations, highlighting the implications of the size and shape of the active doughnut for different types of supported catalysts. Subsequently, we examine six experimental case studies of catalytic oxidation reactions. These cases satisfy two criteria: 1) At least one of the reaction steps is catalysed by the particle and another step is catalysed by the surface, and 2) the catalytic particles are homogeneously dispersed on the surface. Note that this definition excludes catalysis at the particle‐surface interface, for example dual perimeter sites,^11^ which are effectively a single catalyst.

## 1 Pen‐and‐Paper Analysis

### 1.1 Active site or active volume?

When a catalyst is comprised of active particles on an active surface, the classic “active site” concept becomes too simplistic. Instead, different steps of the catalytic cycle occur at different sites. Therefore, we must also consider the interaction between the sites. This interaction can be via chemical communication (exchange of reaction intermediates) and/or via electronic communication (electron transfer between sites in redox catalysis). Thus, the real active site is actually an active *volume*, its borders defined by the intermediates’ lifetime and mobility. Mobility can refer to both mass‐ and electron‐transfer, since active sites may communicate chemically and/or electronically.

**Figure 2:**
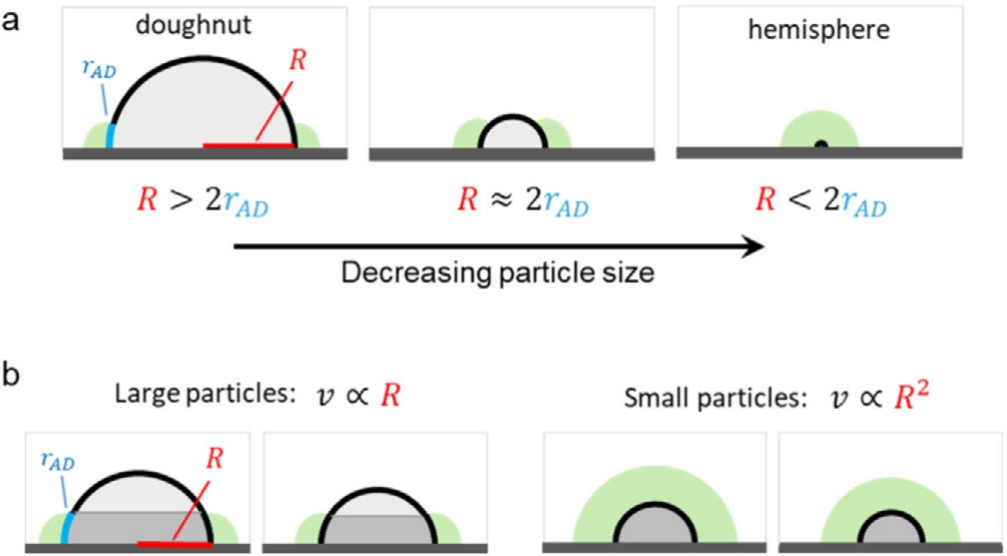
a) The relative size of the active doughnut volume (green) varies with particle size. b) In the case of large particles, the catalytically active surface is a belt circling their base. Thus, the active doughnut radius ($r_{AD}$ ) is proportional to particle radius, and the reaction rate correlates *linearly* to particle size (R ). On small particles, however, the active surface is hemispherical, leading to a *quadratic* correlation.

The shape of the active volume depends on two sets of parameters. First, it depends on the physical characteristics of the catalyst: particle size, shape and dispersion on the surface. For example, if a catalytic particle is large compared with its active volume, this volume is merely a torus at the base of the particle. Conversely, if the particle is small, the active volume may cover it completely, resulting in a hemisphere (Figure 2 a). Second, the dimensions of the active volume are determined by the reactivity, diffusion and sorption of the intermediates. The size of the active volume is directly correlated to the diffusivity and lifetimes of active species. Furthermore, distortions from toroidal/ hemispherical symmetry result from differences in reactivity and sorption between particle and surface.

### 1.2 Practical implications of the active doughnut concept

The active doughnut concept has two important practical implications. First, the reaction rate is no longer proportional to the number of active sites, but rather to the number of “*communicative”* active sites—those available to intermediates during their lifetime. Active sites at the top of a large particle, for example, or at the surface far away from a particle, would be useless for tandem catalysis. If a particle is too large, much of its surface area will go unused. Furthermore, if particles are bunched together, their active volumes overlap, and the catalytic surface is under‐utilized. This has important implications for reporting turnover numbers (TONs), whose values are underestimated when determined in either of these situations. Practically speaking, the most efficient particle‐surface tandem catalysts are those where both particle size and inter‐particle distance are of the same order of magnitude as the active doughnut thickness (*r_AD_*).

Second, the concept generates an important testable prediction, concerning the dependence of the reaction rate (v ) on the particle size (*R*). In most catalytic systems, the total surface area of particles is much smaller than the surface area of the support. This turns particle surface area into a limiting factor in tandem catalysis, yielding an interesting opportunity for testing the “active doughnut” concept experimentally. Consider the following two extreme cases, with particles either much smaller or much larger than their active volumes (as in Figure 2 b). If the particle is small, intermediates can diffuse to its entire surface. In this case the reaction rate (*v*) will depend on the surface area of a *hemisphere* (given by Equation (1), where *A* is the area of the particle that participates in catalysis). Thus, the rate of this reaction step will have a *quadratic* relation to the particle radius: $v\propto R^{2}$ . In contrast, if particles are too large, only a belt‐shaped region surrounding the particle base participates in catalysis, with its area given in Equation (2). In this case, the reaction rate becomes *linearly* dependent on particle radius: v∝R . Note that this behaviour is very similar to that observed for dual perimeter site catalysts, where the activity depends linearly on the particle size.^12^, ^13^ Hence, when gradually increasing particle size, there will be a point where the dependence of v *vs. R* will shift from quadratic to linear. This point can be determined experimentally, and used as an estimate for the effective size of the active doughnut.

(1) $$A_{dome}={2\pi R}^{2}$$

(2) $$A_{belt}=r_{AD}•2\pi R\;$$

## 2 Case Studies

The “active doughnut” idea was first presented in our preliminary communication on catalytic oxidative dehydrogenation of alcohols with molecular oxygen using metal oxide particles on nitrogen‐doped carbon.^10^ In that example, particles were ca. 200 nm in diameter, and spaced about 500 nm apart. The catalyst fulfils both conditions for “active volumes”: the surface and the particles catalyse different reaction steps and the particles are homogeneously dispersed.^10^ Oxidation of alcohols requires the transfer of two protons from the alcohol to the activated oxygen in order to complete the catalytic cycle, forming an aldehyde/ketone and water. This means that both reactions must take place in close proximity, in other words within the active volume.

Here we extend the concept to five additional case studies of different catalytic cycles. These are nickel‐aluminium‐doped hydrotalcite, gold on chromium‐doped hydrotalcite, silver on alumina, platinum on alumina and silver on zinc oxide. They all catalyse the oxidative dehydrogenation of alcohols, either with or without molecular oxygen. Both the particle and the surface (i.e., the support) are catalytically active. The cases are organized by particle size, examining two limiting cases of *R*/*r_AD_*. We start with small particles (≈0.8–3 nm) and then move to large particles (40–200 nm).

### 2.1 Small‐particle catalysts

The groups of Li, Hensen, and Shimizu reported tandem catalysis on ultra‐small particles of Au, Ag, and Pt dispersed on co‐catalytic surfaces of hydrotalcite and alumina.^14^, ^15^, ^16^, ^17^, ^18^

Such small particles (*R*≈0.8–3 nm) set the lowest limit for the *R*/*r_AD_* ratio. Both hydrotalcites (HT) and alumina are important catalytic supports with rich surface chemistry. Hydrotalcites are layered double hydroxides (Mg_6_Al_2_(OH)_16_CO_3_⋅*n* H_2_O), used in oxidation,^19^, ^20^, ^21^, ^22^, ^23^, ^24^, ^25^, ^26^, ^27^, ^28^, ^29^, ^30^ deoxygenation,^31^, ^32^ and dehydrogenation catalysis.^33^, ^34^ Their basicity can be tuned through isomorphic substitution of Mg or Al cations,^35^ and their redox properties by doping with transition metals (especially Cr).^14^ Reducible HT supports help oxidation catalysis by activating O_2_.^36^, ^37^, ^38^ Alumina is a well‐known porous support with both acidic and basic surface sites.^39^, ^40^ The relative proportion of these sites can be tuned by doping the alumina with acidic or basic cations (Mg, Zr, or Si).^15^

**Figure 3:**
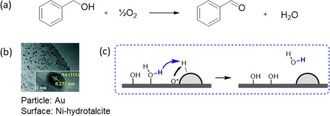
a) Oxidation of benzyl alcohol, catalysed in Li's study by Au/Ni‐Al‐HT. Reaction conditions: 1 mL benzyl alcohol, 10 mg catalyst, 0.1 MPa O_2_, 140 °C, 0.5 h. b) TEM image of Au/NiAl‐HT.^18^ c) The “active doughnut step”: diffusion of a proton from the surface, to recombine with a hydride and an active oxygen species at the particle.

Li's tandem catalysts are based on catalytic gold nanoclusters dispersed on Ni−Al‐doped HT.^18^ The particles’ size is *d* ≈2–3 nm, and they are dispersed at typical inter‐particle distances of 5–15 nm (Figure 3 b). The catalytic mechanism involves the particles, the surface, and the newly formed Au^3+^−Ni^3+^−OH interface. Basic surface groups (e.g., Ni−OH) bind benzyl alcohol to form a Ni‐alkoxide intermediate and release water. The gold particle then closes the catalytic cycle by C−H activation at the Ni‐bound alkoxide, followed by β‐hydride elimination. As the aldehyde product desorbs, a hydride remains bound to the gold particle. In the final step, this hydride is neutralized to water by active oxygen (coming from the Au^3+^−Ni^3+^−OH interface) and a proton diffusing from the surface.

Both particle and surface are catalytically active, yet not every communication between the two creates an active doughnut. For example, the HT‐bound alkoxide is not expected to diffuse through the solution bulk, but rather along the surface, hopping between M^*n*+^−OH groups. Thus, its reaction will be limited to the surface, rather than a volume. Conversely, when a surface proton recombines with the Au‐bound hydride and the active oxygen species (O*) at the Au‐HT interface ring, it can easily diffuse through the solution.

Since the particles are small (≈2.5 nm) and protons diffuse quickly, we expect the active doughnut to cover the particles entirely. Its size will depend on the proton diffusivity in the specific reaction environment—in this case, toluene at 100 °C. Thus, if two of these particles are too close to each other, their active doughnuts will overlap, lowering the effective reaction volume. This emphasises the importance of sufficient particle dispersion.

**Figure 4:**
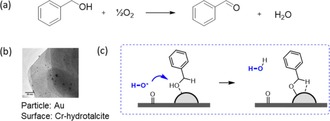
a) Oxidation of benzyl alcohol, as reported by Hensen and co‐workers, catalysed by Au/Cr‐HT. Reaction conditions: 1 mmol benzyl alcohol, 50 mg catalyst, 0.5 mmol *n*‐dodecane (internal standard), 10 mL toluene, 20 mL min^−1^ O_2_, 100 °C, 0.5 h. b) TEM image of Au/Cr‐HT.^14^ c) The “active doughnut step”: OH* produced by O_2_ reduction at the surface, diffuse to the particle‐bound substrate, and dehydrogenate the particle‐bound substrate.

Hensen et al. studied the same reaction, but using a Cr‐doped hydrotalcite as a catalytic surface for supporting gold nanoparticles (*d* ≈0.7–1.3 nm, dispersion 20–50 nm, Figure 4).^14^ Cr doping makes the HT surface redox‐active, allowing it to reduce O_2_ with a helping hand from the Au particle. This occurs by electron transfer from Cr^3+^−OH to the Au, yielding Cr^6+^=O and a free activated oxygen species (OH*).^14^ This species could be a hydroxyl radical or hydroxide anion. Then, benzyl alcohol adsorbs at the gold particle, which activates its hydroxyl group, helped by OH* coming from the surface O_2_ reduction.^41^ The next step is β‐hydride elimination: the aldehyde product desorbs, and a hydride is produced at the Au particle. Finally, the hydride recombines with the Cr‐bound oxygen to form Cr^3+^−OH. The exact mechanism of this recombination is still under debate.

The diffusion of the OH* species from the surface and its recombination at the particle is a reaction step that involves an active doughnut (Figure 4 c). However, if the OH* travels mainly by surface diffusion, the active doughnut will be thin and close to the surface. This situation is probable, since such oxygen species can coordinate easily with surface metal ions. On the other hand, the size of the active doughnut also depends on the electrical conductivity of the support. Since O_2_ activation involves a redox step (oxidation of Cr^III^ by the Au particle), a more conductive substrate could allow this step to occur farther away from the particle. Overall, lacking data on the OH* species and on Cr:HT conductivity, it's hard to estimate the magnitude of these opposing effects—and thus the size of this step's active doughnut.

Interestingly, another reaction step may involve an active doughnut reactive volume. It is the recombination of a particle‐bound hydride with the Cr=O at the surface. However, a hydride adsorbed on a gold surface has a much longer lifetime than one in solution, especially that the solvent (toluene) is apolar. Therefore, bulk diffusion is not expected in this case. In more polar solvents, however, bulk diffusion could be more pronounced, leading to an interaction volume, rather than area.

Zhao et al. have also reported the oxidative dehydrogenation of benzyl alcohol,^42^ this time using Ni‐Al‐layered double hydroxides (Ni‐Al‐LDH) as a catalytic surface, and Au as small (≈5 nm) catalytic nanoparticles. Three separate catalytic regions were proposed: the Ni‐Al‐LDH surface, the Au particle and the interface between Au and the NiO_6_ octahedra. The mechanism is similar to Hensen's: the LDH surface binds the substrate, the Au particle promotes β‐H elimination and the interface layer activates oxygen because of its unique local electronic structure. In contrast to Hensen's hypotheses, these authors suggest that the OH* does not diffuse at all—ruling out an active doughnut scenario.

All of the above cases involved alcohol dehydrogenation, with oxygen as an electron acceptor. All may involve an active doughnut step. The particle sizes are similar (≈1–5 nm), so these cases are still in the “hemisphere”‐type reaction volume (Figure 2 a). The main difference between the cases is in the diffusing species: H^+^ and OH^−^ have different diffusivities and different sorption strengths to the metal hydroxide surface. Thus, the active doughnuts are expected to have different sizes despite the similarities between the systems.

Further differences between the cases stem from the chemical properties of hydrotalcite, which contains many OH^‐^between the HT layers. This renders it basic, and OH^−^‐conductive. Tuning the basicity and OH^−^ conductivity of HT materials may allow changing the active doughnut size. For example, doping may introduce more surface acidic sites, promoting sorption of OH* species (as in Hensen's mechanism). This would reduce surface‐diffusivity and enlarge the active doughnut. However, the electronic conductivity of hydrotalcites is not expected to affect the active doughnut dimensions. Since it requires an activation barrier of 0.5–0.7 eV,^43^ it is only expected to play a role at higher temperatures.

An alternative method for alcohol dehydrogenation, namely without molecular oxygen, was reported by the Shimizu group. Their oxidant‐free dehydrogenation is catalysed by small clusters of silver (0.8–3.0 nm; reaction conditions: 1.0 mmol substrate, 3 mL toluene, 2.0 mol % catalyst, 100 °C, 24 h)^15^ or platinum (1.4 nm; 1.0 mmol substrate, 1 g *o*‐xylene, 0.01 mol % catalyst, 144 °C, 48–90 h)^16^ supported on alumina (Al_2_O_3_).

The particles are spaced tens of nanometres apart, so each particle can accommodate multiple substrate molecules. Both the metal particles and the acid‐base sites on alumina are crucial for high catalytic activity.^44^, ^45^, ^46^ Thus, we may test for active doughnut reaction steps. Shimizu and co‐workers reported quantitative TOF data for different particle sizes.^16^ We then replotted this data based on the total TOF per particle (see Figure S1). While more points are required for a confident conclusion, this data indicates a quadratic relation for small particles and a linear relation for larger particles (assuming this reaction step is slow enough to affect the overall rate).

On both catalysts (Ag/Al_2_O_3_ and Pt/Al_2_O_3_), the alcohol substrate is first deprotonated by the basic, hydroxylated alumina surface. The metal particle then oxidizes this surface‐bound alkoxide to yield an aldehyde product (which desorbs) and a particle‐bound hydride. Finally, a proton diffuses from the surface and recombines with the particle‐bound hydride, and H_2_ desorption completes the catalytic cycle. The last step (H^−^/H^+^ recombination) is expected to occur in the active doughnut volume. The protons are more likely than hydrides to diffuse through solution. Therefore, the acidity of the surface is critical in determining the dimensions of the active doughnut, whereas the nature of the catalytic particle (Ag vs. Pt) is unimportant. Practically speaking, since the active doughnuts on Ag and Pt are similarly sized, switching between the two will not require adjustments regarding particle dispersion.

### 2.2 Large‐particle catalysts

**Figure 5:**
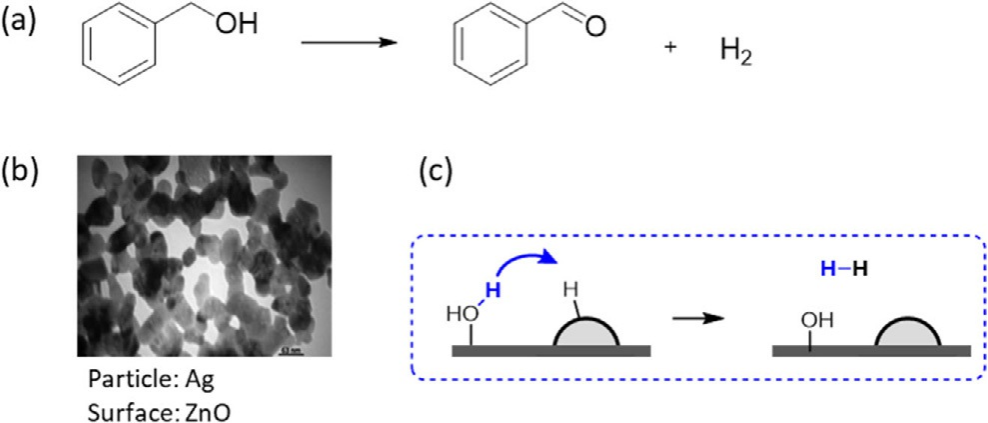
a) Oxidation of benzyl alcohol, catalysed in Hosseini‐Sarvari's study by Ag/ZnO. Reaction conditions: 1 mmol benzyl alcohol, 2 mL toluene, 1 mmol KOH, 5 mg catalyst, 100 °C, 8 h. b) TEM image of Ag/ZnO.^47^ c) The “active doughnut step”: recombination of particle‐bound hydride with a proton diffusing from the surface.

The second category covers those cases where large catalytic particles (*R*≫r_AD_) are supported on catalytic surfaces. Hosseini‐Sarvari and co‐workers prepared Ag/ZnO catalysts for the oxidant‐free dehydrogenation of alcohols (Figure 5).^47^ While this material catalyses the same reaction reported by Shimizu et al., the particles here are much larger (40–50 nm). Moreover, the catalytic substrate is different (ZnO rather than Al_2_O_3_), and a base (KOH) is added to deprotonate the benzyl alcohol substrate. Similarly to Shimizu's catalysts, the active doughnut step here also involves the recombination of proton and hydride. However, the higher *R*/*r_AD_* ratio determines that most of the particles’ surface area remains unused. This is in contrast with Shimizu's materials, where activity is limited by unused surface of the *substrate*. Metal particles by themselves are also known to catalyse dehydrogenation reactions.^48^, ^49^, ^50^ Yet when combined with a metal‐oxide support an alkoxide intermediate can form that is easily oxidized by the metal particle. For large‐particle catalysts, this background reaction of metal‐only catalysed oxidation becomes more important with increasing particle size.

For even larger particles (>100 nm), as in the case of our metal oxide on nitrogen‐doped carbon catalysts,^10^ the active doughnut volume is smaller still, relative to the particle. Here, the nitrogen‐doped carbon surface is responsible for oxygen activation,^51^, ^52^, ^53^ while metal oxide particles (such as CoO_x_ and CuO_x_) catalyse the alcohol oxidation (see illustration in Figure 1). This reaction requires the transfer of two protons from the alcohol to the oxygen. However, as the alcohol is bound on the particle and the oxygen is activated at the surface, the two must be close enough to react within the short life‐span of the active oxygen species. Thus, owing to the large size of the metal oxide particles (≈200 nm), catalysis will occur within a relatively small active doughnut volume at the base of the metal oxide particles.

In summary, we have identified six cases of catalytic oxidation of alcohols where the active doughnut concept applies. These case studies are grouped based on particle size: small (0.8–3 nm), and large (>10 nm). The small‐particle catalysts (Au/Ni‐Al‐HT, Au/Cr‐HT, Pt/Al_2_O_3_, Ag/Al_2_O_3_,) have either alumina or hydrotalcite catalytic surfaces, that contribute acid‐base or redox reactivity. On these supports, the surface active sites are well‐dispersed and active volume is most likely hemispherical. The large‐size category includes the Ag/ZnO catalyst with a particle size of 30–60 nm on a surface with particle spacing of ≈400 nm and the metal‐oxide particles on nitrogen‐doped carbon catalyst with very large particles, spaced micrometres apart. Here the active doughnut volume is much smaller relative to the particles, rendering most of the particles’ surface inactive.

Interestingly, the active doughnut concept could be applied to different reaction mechanisms. Two different mechanisms were proposed for oxidative dehydrogenation with molecular oxygen. In the case of Au/Ni‐Al‐HT, the alcohol is first fixed on the catalytic surface and then reacts on the particle.^42^ Oxygen activation happens at the particle/surface interface. Conversely, in the case of Au/Cr‐HT, the proposed mechanism is that the surface activates oxygen to generate an OH* intermediate that reacts with the substrate which is bound to the particle.^26^ Yet in both cases an activated oxygen species diffuses and recombines with a hydride, creating an active doughnut reaction volume. In acceptorless dehydrogenation, the active doughnut step involves the diffusion of a proton from the catalytic surface to recombine with a hydride on the metal particle.

## 3 Conclusion

When both the particle and substrate are catalytically active, there exists a defined “active doughnut” volume, where most of the catalytic action takes place. Its size and shape can be estimated from the particles’ size and their spatial distribution, as well as from the lifetime, absorption, and diffusivity of the reaction intermediates. The active doughnut concept offers a useful framework for planning and analysing the optimal size of catalytically active particles, and their distribution on the surface. It suggests that calculated turnover frequencies may be underestimated when the particles are too large, or when the particles’ surface concentration is too low. Furthermore, it offers predictive tools: For small particles (*R*≪*r_AD_*), the reaction rate for the ‘active doughnut“ step will have a quadratic dependence on the particle radius, since the whole particle can be utilized. For larger particles, especially when the active doughnut radius is smaller than the particle size (*R*≫*r_AD_*) this dependence will be linear. The transition point between these two correlations (roughly when R≈2r_AD_) may offer a new tool for estimating the size of these active doughnuts without the a priori requirement of a full mechanistic picture. We hope that the framework presented here will cast a new light on the least‐studied reaction steps in catalytic cycles (the fast steps), and ultimately help chemists to design better bifunctional cascade catalysts.

## Conflict of interest

*The authors declare no conflict of interest*.

## Supporting information

As a service to our authors and readers, this journal provides supporting information supplied by the authors. Such materials are peer reviewed and may be re‐organized for online delivery, but are not copy‐edited or typeset. Technical support issues arising from supporting information (other than missing files) should be addressed to the authors.

Supplementary
