# Supplementary material for: Cooperative Surface‐Particle Catalysis: The Role of the “Active Doughnut” in Catalytic Oxidation
Source: ChemCatChem. 2018 Feb 16;10(10):2119–24. doi: 10.1002/cctc.201701819 (PMC6001523; doi:10.1002/cctc.201701819)
Supplement: Supplementary file 1 — Supplementary [file CCTC-10-2119-s001.pdf]

Heterogeneous & Homogeneous & Bio- & Nano-
CHEMCATCHEM
CATALYSIS

Supporting Information

Cooperative Surface-Particle Catalysis: The Role of the "Active Doughnut" in Catalytic Oxidation

Thierry K. Slot,[a] David Eisenberg,*[a, b] and Gadi Rothenberg*[a]

cctc_201701819_sm_miscellaneous_information.pdf

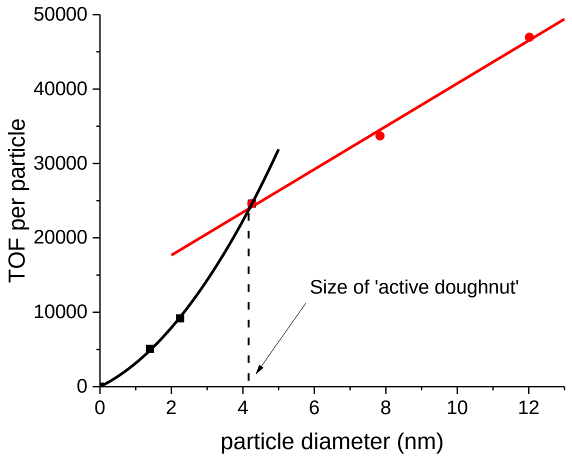

Figure S1: TOF per particle versus particle diameter showing the quadratic to linear-transition for the Pt/Al_2O_3 catalyst reported by Shimizu and co-workers.[1] The data was replotted as TOF per particle (taking the average size as the particle size) and the quadratic curve was fitted through the origin.

[1] K. Kon, S. M. A. Hakim Siddiki, K. Shimizu, *J. Catal.* **2013**, *304*, 63–71.

WILEY-VCH
